# High tibial lateral closing wedge and opening wedge valgus osteotomy produce different effects on posterior tibial slope and patellar height

**DOI:** 10.3389/fsurg.2023.1219614

**Published:** 2023-09-12

**Authors:** Songjie Ji, Yuan Gao, Jun Zhang, Feng Pan, Kunzhi Zhu, Xu Jiang, Yixin Zhou

**Affiliations:** ^1^ Department of Orthopedics, Beijing Jishuitan Hospital, Capital Medical University, Beijing, China; ^2^ Department of Joint Surgery, Beijing Jishuitan Guizhou Hospital, Guiyang, China

**Keywords:** high tibial osteotomy (HTO), open, closed, posterior tibial slope (PTS), patellar height (PH)

## Abstract

**Objective:**

To compare the clinical outcomes of performing a closed tibial high osteotomy with an open osteotomy and the changes in posterior tibia slope and patellar height.

**Methods:**

Methods were collected from three hundred and forty patients (440 knees) with high tibial osteotomy performed from January 2019 to January 2020. Forty patients (50 knees) had a lateral closed wedge tibial osteotomy (LCWHTO), and 300 patients (390 knees) had a medial open wedge tibial osteotomy (MOWHTO). The follow-up periods were 20.5 months and 19.9 months, respectively. At the final follow-up visit, both groups evaluated the Lysholm score and joint range of motion (ROM). Changes in preoperative and postoperative mechanical axis deviation (MAD), proximal medial tibial angle (MPTA), posterior tibial slope (PTS), and M-K index were compared between the two groups of patients.

**Results:**

Lysholm scores were 79.6 ± 15.6 preoperatively and 96.0 ± 5.0 postoperatively in the LCWHTO group (*p* < 0.01); 83.7 ± 16.0 preoperatively and 94.3 ± 9.1 postoperatively in the MOWHTO group (*p* < 0.01). ROM was 136.0° ± 8.4° preoperatively and 133.2° ± 10.1° postoperatively in the LCWHTO group (*p* > 0.05); 136.5° ± 8.4° preoperatively and 135.7° ± 9.3° postoperatively in the MOWHTO group (*p* > 0.05). the MAD was (26.5 ± 4.1) mm preoperatively and 0.3 ± 2.9 mm postoperatively in the LCWHTO group (*p* < 0.01); 21.8 ± 6.5 mm preoperatively and −0.3 ± 2.6 mm postoperatively in the MOWHTO group (*p* < 0.01). The MPTA in the LCWHTO group was 75.3° ± 3.2° preoperatively and 89.5° ± 2.4° postoperatively (*p* < 0.01). 77.1° ± 3.0° preoperatively and 90.6° ± 2.7° postoperatively in the MOWHTO group (*p* < 0.01). M-K index was 0.78 ± 0.08 preoperatively and 0.79 ± 0.07 postoperatively in the LCWHTO group (*p* > 0.05). 0.78 ± 0.05 before and 0.75 ± 0.05 after surgery in the MOWHTO. 10.8° ± 3.0° PTS before and 8.1° ± 3.4° after surgery in the LCWHTO group (*p* < 0.05); 10.2° ± 3.1° preoperatively and 10.9° ± 4.0° postoperatively (*p* > 0.05).

**Conclusions:**

LCWHTO decreases the PTS and has no effect on patellar height; MOWHTO does not affect the PTS but decreases patellar height. The patient should individualize the choice of the osteotomy.

## Introduction

High tibia osteotomy (HTO) was the most successful and commonly used for treating medial compartment osteoarthritis of the knee with varus malalignment (1, 2). The basic concept of HTO was a correction of coronal malalignment, thereby shifting the weight-bearing load from the injured area to an uninjured area. The pressure in the medial compartment is decompressed, resulting in pain-relieving and delaying cartilage damage; even partial cartilage regeneration can be observed (3). In the past, lateral closed wedge HTO (LCWHTO) was the most widely used procedure; however, medial open wedge HTO (MOWHTO) was predominantly used recently owing to several advantages (4). The two types of osteotomy have their characteristics, with differences in the accuracy of lower extremity mechanical axis correction, postoperative effects on patellar height (PH), and posterior tibia slope (PTS). Changes in the PH and PTS may result in patellofemoral osteoarthritis and anterior cruciate ligament (ACL) injuries due to increased retro patellar cartilage pressure and higher anteroposterior translation, respectively. Previous studies have suggested that LCWHTO increases PH and decreases PTS, whereas MOWHTO decreases PH and PTS (5). However, most previous studies have a small sample size and inaccurate measuring methods (6, 7). This study aimed to compare the differences between these two methods by multi-center data through clinical evaluation and radiographic measurements.

## Materials and methods

From January 2019 to January 2022, all patients undergoing HTO in Beijing Jishuitan Hospital, Guizhou provincial orthopedics hospital, Beijing Chaoyang emergency medical and rescue center, and Shenyang Jishuitan Hospital were enrolled in this retrospective study. This study obtained written informed consent from participants or their guardians and was approved by the Beijing Jishuitan Hospital Institutional Review Board for retrospective data analysis. The inclusion criteria were as follows: (1) patients with symptomatic genu varus, (2) patients in good condition for ligaments of the knee, and MRI was performed to check it if necessary, (3) active mobility is greater than 100° by physical examinations, (4) medial proximal tibia angle (MPTA) was less than 83°, (5) mechanical axis deviation (MAD) was greater than 8 mm. The Exclusion criteria included (1) lateral and patellofemoral compartments symptoms and (2) rheumatoid arthritis or inflammatory arthritis. All patients were fixed with TomoFix® plates (Johnson & Johnson, New Jersey, USA), LCWHTO with TomoFix® proximal tibial lateral anatomic locking plates, and MOWHTO with TomoFix® proximal tibial medial anatomic locking plate.

## Surgical procedure

All patients were designed preoperatively using the Miniaci method (8). Intraspinal anesthesia was used, a tourniquet with a pressure of 300 mmHg was used during the procedure, a tourniquet was released to stop bleeding before closing the wound, and drainage was left at the osteotomy site. (1) LCWHTO: a curved oblique skin incision from 2 cm below the lateral joint line. Before tibial osteotomy, fibular midshaft osteotomy was performed at about 15 cm distal to the fibular head to decrease its tethering effect. A Kirschner pin was inserted parallel to the articular surface 2 cm below the joint line for tibial osteotomy. The calculated wedge length was marked; a second Kirschner pin was inserted toward the medial side. Osteotomy was performed to preserve the medial cortex under fluoroscopic guidance. A wedge-shaped bone was removed, leaving the medial cortex and periosteum intact. Using the creep phenomenon, a valgus force was gradually applied to close the osteotomy site. After the target mechanical axis was confirmed by fluoroscopy, the osteotomy site was fixed with a Tomofix plate. The fibular is drilled and tied with absorbable thread. (2) MOWHTO: a longitudinal skin incision was made in the medial aspect of the proximal tibia. The pes anserine was cut partially, and the superficial medial collateral ligament (MCL) was released. Distal to the joint line (3.5 cm) (9), 2 Kirschner pins were passed from the metaphyseal-diaphyseal junction in the direction of the hinge point between the tip and circumference line of the fibular head. Horizontal osteotomy was performed, leaving the lateral cortex intact. Afterward, an additional coronal osteotomy was performed almost parallel to the coronal plane and at an approximate angle of 110° with the horizontal osteotomy line. The osteotomy site was gradually opened to the calculated wedge length, and the target mechanical axis was confirmed by fluoroscopy. The osteotomy site was fixed with a Tomofix plate, and bone grafting was performed using calcium sulfate (CaSO4)/calcium phosphate (CaPO4) composite (PRO® DENSE Wright Medical Technology, Arlington, TN USA). The drainage was removed in 24 h, and partial weight-bearing was achieved under the protection of a double crutch in 48 h. Partial weight-bearing with crutches was allowed for six weeks. If the osteotomy was healing well, full weight-bearing was encouraged. They were reviewed at six weeks, 1-year, and 2-year postoperatively. Anteroposterior and lateral and lower limbs full-length films were taken.

## Clinical and radiographic evaluation

**Figure 1:**
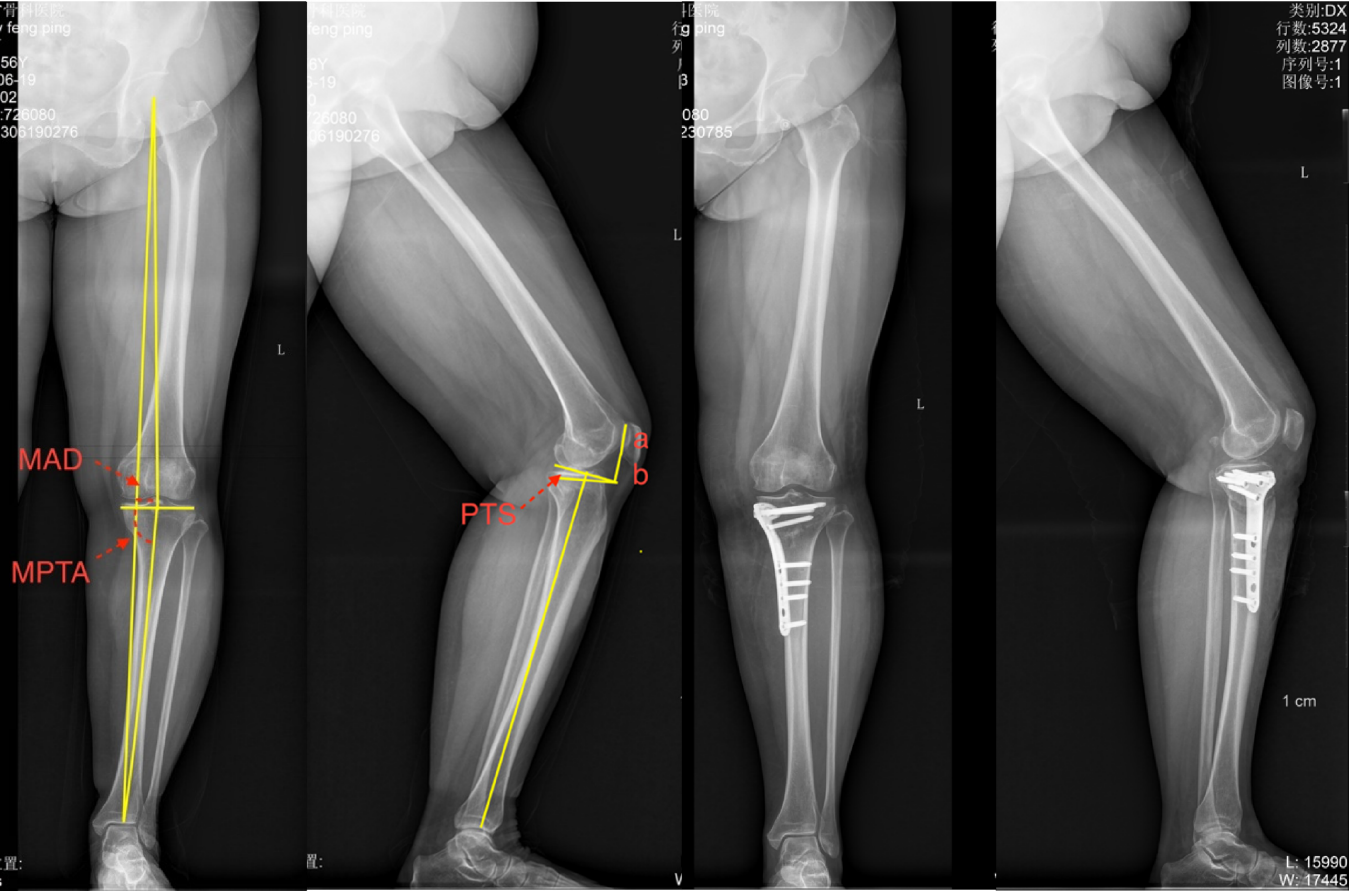
Preoperative and post-operative lower limb full-length, AP, and lateral films of the knee. MPTA: medial angle between the tangent line of the tibial plateau and the tibial mechanical axis in the coronal plane; MAD: distance from the center of the knee to the mechanical axis of the lower limb; PTS: angle between the vertical line of the tibial mechanical axis and the tangent line of the tibial plateau in the sagittal plane; M-K index: ratio of the distance from the lower edge of the patella to the tangent line of the femoral condyle to the length of the medial surface of the patella (a/b).

All patients' knee conditions were clinically evaluated preoperatively and at follow-up using the Lysholm score pre- and post-operative last follow-up. The evaluation included a ROM and Lysholm knee score (10). Independent of the surgical team, a rehabilitation doctor performed these measurements and was blinded to the radiographic findings. The radiographic examination used a digital fluoroscopy system (Sonial-vision Saline lI, Shimadzu, Japan). Pre- and post-operative lower extremity full-length radiographs of the patient's lower limbs and AP and lateral radiographs of the knee were taken (Figure 1). Changes in the patient's pre- and post-operative MPTA, MAD, and PTS were measured. The M-K index (Miura-Kawamura index) is estimated to estimate PH. The measurement method is shown in Figure 1.

## Statistical analysis

Two experienced orthopedic surgeons collected all radiographic parameters and repeatedly assessed them in a blinded fashion one week after the first measurement to reduce bias. The interclass correlation coefficients for the intra- and inter-observer agreements were calculated. Statistical evaluation was performed using SPSS 26.0 (IBM Corp., Armonk, NY, USA). The continuous data are presented as mean values ± standard deviation (SD). Differences between the groups were analyzed with the student-t test for continuous variables and the Pearson chi-square test for categorical variables. Differences were considered statistically significant at *p* < 0.05.

## Results

From January 2019 to January 2022, 40 cases (50 knees) underwent LCWHTO, and 300 patients (390 knees) underwent MOWHTO were included. The two groups were compared, including gender, age, body mass index (BMI), preoperative range of motion (ROM), Lysholm score, M-K index, PTS, and other information. There was no statistically significant difference (*P* > 0.05). The MPTA and mechanical axis deviation (MAD) were compared between the LCWHTO and MOWHTO groups (*P* < 0.05) (Table 1).

**Table 1:** Demographics characteristics, preoperative clinical evaluation, and radiographic measurement of LCWHTO and MOWHT.

	LCWHTO	MOWHTO	*t/χ*2	*p*
Case/knee	40/50	300/390	N/A	N/A
Male/female	10/30	84/216	0.159	0.690
Left/right	28/22	225/165	0.652	0.820
Age	49.2 ± 8.2	50.4 ± 8.5	−0.954	0.341
BMI	26.5 ± 4.8	26.7 ± 3.2	−0.583	0.560
Lysholm score	79.6 ± 15.6	83.7 ± 16.0	−1.687	0.092
ROM (°)	136.0 ± 8.4	136.5 ± 8.4	−0.431	0.067
MPTA (°)	75.3 ± 3.2	77.1 ± 3.0	−4.007	0.000
MAD (mm)	26.5 ± 4.1	21.8 ± 6.5	5.026	0.000
PTS (°)	10.8 ± 3.1	10.2 ± 3.1	1.250	0.212
M-K index	0.78 ± 0.08	0.78 ± 0.05	−0.021	0.983

The Lysholm score for LCWHTO was improved from 79.6 ± 15.6 to 96.0 ± 5.0 (*p* = 0.000); the Lysholm score for MOWHTO improved from 83.6 ± 16.0 preoperatively to 94.3 ± 9.1 postoperatively (*p* = 0.000). The postoperative improvement over preoperative was statistically significant in both groups. 133.2° ± 10.1° of ROM after LCWHTO and 135.7° ± 9.3° of ROM after MOWHTO, with no statistically significant difference between the two groups compared to preoperative ROM. There were no infections, vascular nerve complications, or postoperative complications such as non-union of the bone in either group. A comparison of the parameters between the two groups is shown in Table 2.

**Table 2:** Comparison of various parameters between the two groups after surgery.

	LCWHTO	MOWHTO	*t*	*p*
Follow-up (month)	20.8 ± 5.7	18.9 ± 5.3	2.395	0.017
Lysholm	96.0 ± 5.0	94.3 ± 9.1	0.224	0.823
ROM (°)	133.2 ± 10.1	135.7 ± 9.3	−1.772	0.077
MPTA (°)	89.5 ± 2.4	90.6 ± 2.7	−2.086	0.005*
MAD (°)	0.3 ± 2.9	−0.3 ± 2.6	1.743	0.082
M-K index	0.79 ± 0.07	0.75 ± 0.05	4.262	0.008*
PTS (°)	8.1° ± 3.4°	10.5 ± 3.1	−4.802	0.000*

*p < 0.05, significant difference.

The postoperative MAD was (0.3 ± 2.9) mm in the LCWHTO group and (−0.3 ± 2.6) mm in the MOWHTO group. Both two groups were a statistically significant difference compared with that of the preoperative, respectively. The postoperative MPTA was 89.5° ± 2.4° in the LCWHTO group and 90.6° ± 5.0° in the MOWHTO group. The postoperative M-K index in the LCWHTO group was 0.75 ± 0.05. The postoperative PTS in the LCWHTO group was 0.79 ± 0.07, with a statistically significant decrease compared with that of the preoperative; the postoperative PTS in the MOWHTO group was 10.9° ± 4.0°, with no difference compared with the preoperative. The detail of comparisons preoperative and postoperative between the two groups are shown in Table 3.

**Table 3:** Comparison of preoperative and postoperative radiographic evaluation between the two groups.

	MPTA (°)		MAD (mm)		M-K index		PTS (°)	
	Pre-op	Post-op	Pre-op	Post-op	Pre-op	Post-op	Pre-op	Post-op
LCWHTO	75.3 ± 3.2	90.6 ± 2.7	26.5 ± 4.1	0.3 ± 2.9	0.78 ± 0.08	0.79 ± 0.07	10.8 ± 3.0	8.1 ± 3.4
*t*	−24.872		5.084		−0.573		4.185	
*p*	0.004		0.000		0.568		0.000	
MOWHTO	77.1 ± 3.0	94.3 ± 9.1	21.8 ± 6.5	−0.3 ± 2.6	0.78 ± 0.05	0.75 ± 0.05	10.2 ± 3.1	10.5. ± 3.1
*t*	−64.554		62.753		7.404		−1.451	
*p*	0.000		0.000		0.000		0.147	

## Discussion

Coventry started performing LCWHTO in 1965. The advantages of LCWHTO were good initial stability, no need for bone grafting, and a lower rate of non-union. The disadvantages are the need for a concomitant fibular osteotomy or superior tibiofibular joint separation and the potential for an axial offset of the proximal tibia after the osteotomy, which complicated total knee replacement if it was needed. The risk of common peroneal nerve injury is high (incidence of 1% to 2.8%). In recent years, MOWHTO has become more popular due to innovations in internal fixation materials, fixation techniques, and a wider choice of materials for bone grafting (11). The advantages of MOWHTO were that the procedure was easy to control and more accurate, with no need for a fibular osteotomy, has a low risk of common peroneal nerve injury, does not shorten the length of the lower limb, and less soft tissue stripping. The disadvantages are that bone grafting may be necessary, and there is a relatively high risk of delayed or non-healing of the bone. There may also be an increased PTS, decreased patellar height, and increased patellofemoral joint cavity pressure. MOW and LCW techniques allow for high return rates to work and sport. Ekhtiari illustrated return to sport and work rates of 87% and 85%, respectively (12). In the present study, there was no difference in the postoperative Lysholm score and ROM between the two groups at a mean follow-up of 20.5 months and 19.9 months, and both showed significant improvement compared to the preoperative period. Thus, MOWHTO and LCWHTO were essential techniques to consider candidates with genu vara who wish to return to sports, especially those involving impact loading. The most common alternative to HTO is medial unicompartmental knee arthroplasty, which has been show in some studies to be equally effective in restoring patients' activity levels (13, 14). However, unlike unicompartmental knee arthroplasty, HTO allows a faster return to impact work and sporting activities at a higher rate (15).

Recent studies have shown that OWHTO has several advantages over CWHTO, including higher accuracy of correction, better survival at ten years, a more comprehensive range of motion, less soft-tissue dissection, and more reserve of the proximal tibioﬁbular joint (16). However, OWHTO also increases the PTS and limb length and decreases the PH (17) Change in PTS was a risk factor for rupture of the ACL and PCL (18, 19). Therefore HTO should avoid PTS changes as possible. This study showed a difference in the preoperative MAD (*p* = 0.013) and MPTA (*p* = 0.000) between the two groups, with LCWHTO being more severe than the MOWHTO varus groups. This suggests that surgeons prefer LCWHTO in more severe cases. Matsuda compared the PTS between the varus of the knee (9.9°) and the normal knee (10.7°), and there was no difference between the two groups. Our study, therefore, suggested that the change in PTS after surgery was independent of the extent of genu varus.

Most previous studies have concluded that LCWHTO would decrease PTS and MOWHTO would increase PTS (20). A meta-analysis by Nha (21) summarised previous studies and concluded that LCWHTO decreased PTS and MOWHTO increased PTS. The increase in tibial posterior slope is attributed to the perpendicular orientation of the lateral tibial cortex, incomplete osteotomy of the posterolateral cortex, and incomplete release of posterior soft tissue (22). There are many methods to measure PTS, the tibial proximal anatomical axis (TPAA) is not affected by the patient's gender, age, or weight and is the most constant (23), so this study used TPPA for PTS measurements. In this study, the LCWHTO group had a statistically significant decrease in PTS postoperatively compared with preoperatively (*p* = 0.011). In contrast, there was no statistically significant difference in the change in PTS in the MOWHT group preoperatively and postoperatively (*p* = 0.194). This is different from many previous reports in the literature. The statement suggests that by paying attention to the operating points during the procedure, MOWHTO can prevent an increase in PTS. This indicates that careful management of the surgical technique and proper positioning of the components can help avoid undesired changes in the posterior tilt during the procedure.

The change in PTS was associated with several reasons. At first, the proximal tibia was like a triangular column, anteriorly narrow and posteriorly wide. So the osteotomy plane was angled with the coronal plane. The bottom edge of the wedge-shaped bone block resected in LCWHTO was located anterolaterally rather than laterally to the tibia, resulting in more anterior bone removal than posterior and decreased PTS, whereas, in MOWHTO, the osteotomy gap was located anteromedially to the tibia, with more spreading anteriorly than posterior, which increases PTS This increases PTS. Secondly, the origin of the superficial MCL on the tibial side was on the posteromedial side of the proximal tibia. For MOWHTO spreading, more posterior resistance resulted in a narrower spreading gap than anteriorly (24). Thirdly, for MOWHTO, the anteromedial tibia was exposed much better than the posteromedial tibia due to the anteromedial incision and simple anteromedial anatomy. Tomofix plate can easily be placed anteriorly.LaPrade's study showed that PTS increased by 4.3° when the plate was placed anteriorly and by only 1° when the plate was placed posteriorly (25). Therefore, we have several key points to avoid increased PTS when performing MOWHTO. At first, care should be taken to place the spreader as far posteriorly as possible so that the posterior spreading gap can be slightly more than the anterior gap, with a slightly trapezoidal gap to counteract error caused by the angle between the osteotomy plane and the coronal plane. The pes anserine and the superficial MCL should be adequately released. Otherwise, the gap would be wide anteriorly and narrow posteriorly, increasing the PTS.

Secondly, when placing the internal fixation, the plate should be placed as far posteriorly as possible on the medial side of the tibia rather than on the anteromedial of the proximal tibia (26). Application of the plate in a more posterior position not only avoid changes to PTS but also provides greater stability (26). Thirdly, after intraoperative fluoroscopic examination of the coronal mechanical axis, it was essential to determine the PTS on fluoroscopy before fixation. The greater the extent of correction, the more care must be taken. Otherwise, the greater the effect on the PTS. Schubert also emphasized the necessity for a small anteroposterior gap in his study of MOWHTO, where the postoperative PTS increased by only 0.07°, a statistically insignificant increase compared with the preoperative (27). The increase in PTS in MOWHTO was also shown to be avoidable in our study.

Previous studies have suggested that MOWHTO decreases PH and LCWHTO increases PH. Insall-Salvati, Blackburn-Peel, and Caton-Deschamps parameters PH were commonly used to measure patellar height. However, HTO changes the proximal tibial anatomy, and using femoral anatomical landmarks as a reference would be more accurate. Hence the M-K index was used for measurement in this study (28). This study showed no statistically significant change in PH after LCWHTO compared with preoperatively (*p* = 0.726). There was a statistically significant difference in PH after MOWHTO compared with preoperatively (*p* = 0.000). The absence of an increase in PH in the LCWHTO group was probably due to the osteotomy position being far from the joint line, which was through the lower part of the tibial tuberosity, leaving the patellar tendon origin intact and minimizing the effect on the knee extension device. Miura concluded that a change of less than 10% in the patellofemoral position would have no impact on the function of the knee extension device. In this study, the M-K index in the MOWHTO group decreased by 3% postoperatively compared with the preoperative and had little effect on clinical function.

## Conclusion

LCWHTO and MOWHTO were effective procedures for unloading the medial compartment of the knee joint. Satisfactory clinical results were obtained with both LCWHTO and MOWHTO.MOWHTO avoids an increase in PTS by improving surgical procedures. MOWHTO should be avoided in patients with preoperative patellar baja to prevent further aggravation of the patellar baja. LCOHTO may decrease the PTS and should be chosen cautiously in cases with a minor preoperative PTS.

## Data Availability

The original contributions presented in the study are included in the article/Supplementary Material, further inquiries can be directed to the corresponding authors.
